# A Deep Learning Approach for Predicting Antidepressant Response in Major Depression Using Clinical and Genetic Biomarkers

**DOI:** 10.3389/fpsyt.2018.00290

**Published:** 2018-07-06

**Authors:** Eugene Lin, Po-Hsiu Kuo, Yu-Li Liu, Younger W.-Y. Yu, Albert C. Yang, Shih-Jen Tsai

**Affiliations:** ^1^Department of Electrical Engineering, University of Washington, Seattle, WA, United States; ^2^Graduate Institute of Biomedical Sciences, China Medical University, Taichung, Taiwan; ^3^Department of Public Health, Institute of Epidemiology and Preventive Medicine, National Taiwan University, Taipei, Taiwan; ^4^Center for Neuropsychiatric Research, National Health Research Institutes, Miaoli County, Taiwan; ^5^Yu's Psychiatric Clinic, Kaohsiung, Taiwan; ^6^Department of Psychiatry, Taipei Veterans General Hospital, Taipei, Taiwan; ^7^Division of Psychiatry, National Yang-Ming University, Taipei, Taiwan; ^8^Division of Interdisciplinary Medicine and Biotechnology, Beth Israel Deaconess Medical Center/Harvard Medical School, Boston, MA, United States; ^9^Institute of Brain Science, National Yang-Ming University, Taipei, Taiwan

**Keywords:** antidepressant, deep learning, genome-wide association studies, major depressive disorder, multilayer feedforward neural networks, personalized medicine, single nucleotide polymorphisms

## Abstract

In the wake of recent advances in scientific research, personalized medicine using deep learning techniques represents a new paradigm. In this work, our goal was to establish deep learning models which distinguish responders from non-responders, and also to predict possible antidepressant treatment outcomes in major depressive disorder (MDD). To uncover relationships between the responsiveness of antidepressant treatment and biomarkers, we developed a deep learning prediction approach resulting from the analysis of genetic and clinical factors such as single nucleotide polymorphisms (SNPs), age, sex, baseline Hamilton Rating Scale for Depression score, depressive episodes, marital status, and suicide attempt status of MDD patients. The cohort consisted of 455 patients who were treated with selective serotonin reuptake inhibitors (treatment-response rate = 61.0%; remission rate = 33.0%). By using the SNP dataset that was original to a genome-wide association study, we selected 10 SNPs (including *ABCA13* rs4917029, *BNIP3* rs9419139, *CACNA1E* rs704329, *EXOC4* rs6978272, *GRIN2B* rs7954376, *LHFPL3* rs4352778, *NELL1* rs2139423, *NUAK1* rs2956406, *PREX1* rs4810894, and *SLIT3* rs139863958) which were associated with antidepressant treatment response. Furthermore, we pinpointed 10 SNPs (including *ARNTL* rs11022778, *CAMK1D* rs2724812, *GABRB3* rs12904459, *GRM8* rs35864549, *NAALADL2* rs9878985, *NCALD* rs483986, *PLA2G4A* rs12046378, *PROK2* rs73103153, *RBFOX1* rs17134927, and *ZNF536* rs77554113) in relation to remission. Then, we employed multilayer feedforward neural networks (MFNNs) containing 1–3 hidden layers and compared MFNN models with logistic regression models. Our analysis results revealed that the MFNN model with 2 hidden layers (area under the receiver operating characteristic curve (AUC) = 0.8228 ± 0.0571; sensitivity = 0.7546 ± 0.0619; specificity = 0.6922 ± 0.0765) performed maximally among predictive models to infer the complex relationship between antidepressant treatment response and biomarkers. In addition, the MFNN model with 3 hidden layers (AUC = 0.8060 ± 0.0722; sensitivity = 0.7732 ± 0.0583; specificity = 0.6623 ± 0.0853) achieved best among predictive models to predict remission. Our study indicates that the deep MFNN framework may provide a suitable method to establish a tool for distinguishing treatment responders from non-responders prior to antidepressant therapy.

## Introduction

Personalized medicine, an emerging paradigm of medicine, is developing into the cornerstone of healthcare practice in terms of medical decisions and treatments tailored to the individual patient (1, 2). More precisely, patients are partitioned into subgroups by genetic and clinical characteristics, thereby medications could be tailored to specific patients with comparable genetic and clinical biomarkers (3). More broadly, personalized medicine promises to offer accurate diagnostic and therapeutic approaches in a patient-specific manner during all stages of patient care, including prevention, diagnosis, prognosis, treatment, and follow-up (4). The usage of genetic and clinical biomarkers has highlighted a key role in personalized medicine in the field of chronic diseases such as psychiatric or mental disorders (5, 6). Although the integration of personalized medicine into clinical decision making is still emerging, symbolic progress has recently been made by using genetic and clinical information to facilitate better predictions of patients' responses to targeted therapy (7). For instance, several genome-wide association studies (GWAS) have been carried out to pinpoint susceptible genetic loci influencing antidepressant treatment response as an entity (5, 6, 8). Moreover, accumulating evidence implicates that carefully chosen single nucleotide polymorphisms (SNPs) could be utilized as genetic biomarkers to infer clinical treatment outcomes and adverse drug reactions in patients with major depressive disorder (MDD) treated with antidepressants (5, 6, 8).

Recent advances in deep learning have demonstrated its power to learn and recognize complex non-linear hierarchical patterns based on large-scale empirical data (9). In general, the objective of deep learning is to facilitate an algorithm to learn a hierarchical representation of the data via multiple layers of abstraction such as multi-layer feedforward neural networks (MFNNs) (10). Due to new techniques such as the deployment of General-Purpose Computing on Graphics Processing Units, deep learning has carried out state-of-the-art performances on a wide variety of applications such as molecular biology (11). In the generic terms, the workflow for a deep learning algorithm is comprised of three portions including the model building from example inputs, evaluation and tuning of the model, and then the model production in prediction-making (12). In other words, a deep learning algorithm for classification applications such as medical diagnosis in personalized medicine is a procedure for choosing the best hypothesis from a set of alternatives that fit a set of observations (13).

The use of personalized medicine in terms of predicting antidepressant treatment response is still in its infancy. Scant human studies have investigated methods to build prediction models for estimating antidepressant treatment response. A study by Kautzky et al. suggested that a random forest prediction model for treatment outcome correctly identified 25% of responders by using 3 SNPs (including *BDNF* rs6265, *PPP3CC* rs7430, and *HTR2A* rs6313) and a clinical variable (that is, melancholia) (14). The following study by Patel et al. reported that an alternating decision tree model estimated treatment response with 89% accuracy by using structural imaging, age and mini-mental status examination scores (15). Moreover, another study by Chekroud *et al*. implicated that a machine learning model predicted clinical remission by using 25 variables with 59% accuracy (16). Iniesta et al. also demonstrated that regularized regression models based on clinical and demographical characteristics can predict response with clinically meaningful accuracy (17). Finally, a recent study by Maciukiewicz *et al*. showed that a support vector machine model forecasted treatment response with 52% accuracy by using SNPs (18).

In light of the aforementioned considerations, we hypothesized that it could be feasible and effective to use deep learning to build predictive models of antidepressant treatment outcome. To the best of our knowledge, no previous studies have been performed to evaluate predictive models for drug efficacy of antidepressants by using deep learning techniques. First, we explored for susceptibility loci by conducting a GWAS study with antidepressant treatment response *per se* in a hypothesis-free manner. Then, we combined genetic and clinical variables to optimize prediction of antidepressant treatment outcome using deep MFNN models. We selected the deep MFNN models because these models can be commonly utilized to solve complex applications in classification and predictive modeling and these models possess the benefits of fault tolerance, non-linearity, integrality, and real-time operations (19, 20).

## Materials and methods

### Study population

The study cohort is comprised of 455 patients, 268 patients from NHRI (The National Health Research Institutes) and 187 patients from TVGH (Taipei Veterans General Hospital), who were diagnosed with MDD in two central institutes in Taiwan. Subjects were part of the International SSRI Pharmacogenomics Consortium (ISPC) project encompassing 7 member sites from 5 countries (21). The diagnosis was assessed by board-certified psychiatrists who interviewed outpatients and obtained medical records. The inclusion criteria were that (1) subjects were Taiwanese with a minimum baseline score of 14 on the 21-item Hamilton Rating Scale for Depression (HRSD), and (2) either subjects were first-episode cases or had withdrawal of antidepressants for more than 2 weeks prior to entry into the study. Exclusion criteria were additional current DSM-IV Axis I diagnoses (including substance abuse, generalized anxiety disorders, panic disorders, or obsessive compulsive disorders), personality disorders, pregnancy, recent suicide attempt, and major medical and/or neurological disorders. These patients were treated with selective serotonin reuptake inhibitors (SSRIs), which include escitalopram (38.5%), paroxetine (38.5%), fluoxetine (18.3%) and citalopram (4.8%). Patients were assessed repeatedly at baseline and week 2, 4, and 8 using the 21-item HRSD.

Experiments were conducted in accordance with the Declaration of Helsinki and approved by the Institutional Review Board of Taipei Veterans General Hospital (VGHIRB No.: 2014-06-001B). Written informed consent was obtained from all participants ensuring adequate understanding of the study.

### Measurement

Measurements of treatment response were obtained for participants as follows (22). First, we measured the sum score of 21-item HRSD at the 8th week of antidepressant treatment and recoded the results as “*non-remitted*” if the sum score was greater than 7 and as “*remitted*” otherwise. Second, we measured the percentage change of HRSD (that is, %ΔHRSD) and recoded the results as “*non-response*” if the percentage change was greater than −50% and as “*response*” otherwise.

### Genotyping data and quality controls

For all participants, SNP genotyping was carried out using Illumina HumanOmniExpressExome BeadChips in the International SSRI Pharmacogenomics Consortium. A total of 455 subjects were genotyped with 951,123 SNPs.

First, we performed quality control procedures with each individual, including kinship, sample quality, and population stratification (23). Then, plate-wise genotyping biases were checked. Samples with plate pass rate greater than 97% were retained for the subsequent analyses. We removed a total of 18 samples (11 from NHRI and 7 from TVGH) during this step. Secondly, the inbreeding coefficient and identity by state (IBS) were examined, and thereby we eliminated samples with strong kinship. In total, 9 subjects (4 from NHRI and 5 from TVGH) were removed due to the similarity measures far away from the clustering (that is, outliers in terms of the IBS distance). Thirdly, a multidimensional scaling analysis method was utilized with the genome-wide IBS pairwise distance to eliminate outliers. Our results showed that none was away from the clustering on the scatter plot. Finally, 7 patients treated with sertraline (SSRI) and venlafaxine (serotonin and norepinephrine reuptake inhibitor) were excluded. As a result, 421 MDD patients were retained for the subsequent analyses.

In addition, we performed quality control procedures as follows for SNP exclusion from further analyses (24). We removed SNPs which failed the Hardy-Weinberg tests with a *P*-value less than 0.0001, genotype missing rate greater than 5%, minor allele frequency (MAF) smaller than 0.05, or bad calling ones in clustering (for example, heterozygous genotypes were falsely called as homozygous, or homozygous genotypes were incorrectly called as heterozygous). After performing the aforementioned quality control procedures, a total of 647,030 SNPs in the samples were retained for the subsequent imputation analysis. The genotyping call rate was 99.9% for all subjects.

### Imputation

Imputation was carried out using IMPUTE2 v3 (25), with haplotype reference panels (https://mathgen.stats.ox.ac.uk/impute/data_download_1000G_phase1_integrated_SHAPEIT2.html) released in March/April 2012 from the 1000 Genomes Project on the basis of HapMap build 37. Only imputed SNPs with high genotype information contents (that is, IMPUTE information score > 0.5) were used in the subsequent association analyses. In total, 30,040,257 SNPs were imputed with high confidence for each individual in the samples. Then, we removed markers which failed the Hardy-Weinberg tests with a *p*-value less than 0.0001, genotype missing rate greater than 5%, MAF smaller than 0.05, or bad calling ones in clustering. As a result, a total of 4,241,701 SNPs were retained for the subsequent analyses.

### Statistical analysis

The Student's *t*-test was conducted to measure the difference in the means of two continuous variables. We performed the chi-square test for categorical data. In order to evaluate the relation of the investigated SNPs with antidepressant treatment outcome, we conducted a logistic regression analysis to evaluate the odds ratios (ORs) and their 95% confidence intervals (CIs), adjusting for covariates including age, sex, and site (26). Multiple testing was adjusted by the Bonferroni correction. The criterion for significance was set at *P* < 0.05 for all tests. Data are presented as the mean ± standard deviation.

To investigate SNP-SNP interactions, we leveraged the generalized multifactor dimensionality reduction (GMDR) method (27). We tested two-way interactions using 10-fold cross-validation. The GMDR software provides some output parameters, including the testing accuracy and empirical *P*-values, to assess each selected interaction. Moreover, we provided age, sex, and site as covariates for SNP-SNP interaction models in our interaction analyses. Permutation testing obtains empirical *P*-values of prediction accuracy as a benchmark based on 1,000 shuffles.

### Predictive model algorithms

In this study, we employed a key deep learning technique, MFNNs. Logistic regression analysis, the standard method for clinical classification (10), was employed to compare with the MFNN models. The Waikato Environment for Knowledge Analysis (WEKA) software (10) was utilized to perform these predictive models.

An MFNN model consists of one input layer, one or multiple hidden layers and one output layer. The MFNN model is one category of artificial neural network algorithms where networks between entities construct no directed cycles (28). In other words, a loop or cycle does not occur in the network because the data only relays in an onward direction from the input neuron panel, through various panels of the hidden neuron portions (if any), and then to the output neuron panel.

Moreover, the principal operation of the MFNN model is subdivided into the learning and retrieving stages in terms of an algorithmic point of view (19). In the learning stage of the MFNN model, the back-propagation algorithm (29) is leveraged for the learning strategy. Additionally, in the retrieving stage, the MFNN model repeats via all the panels to carry out the retrieval process at the output panel in keeping with the inputs of test patterns. On the other hand, from a structural point of view, the MFNN model is an iterative and spatial neural network that possesses various panels of hidden neuron portions among the input and output neuron panels (19).

Here, to train the MFNN models, WEKA's parameters were chosen as follows: the momentum = 0.01, the learning rate = 0.001, the batch size = 100, and the number of epochs = 500.

### Evaluation of the predictive performance

The receiver operating characteristic (ROC) methodology was employed and the area under the ROC curve (AUC) was calculated to evaluate the performance of predictive models (30–32). The AUC of a predictive model can be viewed as the probability that the predictive model will rank a randomly selected positive sample higher than a randomly selected negative one (32). Because AUC is a better performance metric than accuracy, most researchers have adopted AUC for estimating predictive capability of predictive models nowadays (32). The better the prediction model, the higher the AUC (30, 32). In the present work, we utilized AUC to evaluate the performance of various prediction models on a dataset. Additionally, sensitivity (i.e., the proportion of correctly predicted responders of all tested responders) and specificity (i.e., the proportion of correctly predicted non-responders of all the tested non-responders) were measured.

Moreover, the repeated 10-fold cross-validation method was employed to investigate the generalization of the predictive algorithms generated by the aforementioned models (30, 33). Firstly, we randomly split the whole dataset into 10 separate segments. Secondly, in order to evaluate the predictive performance, we trained the predictive model using nine-tenths of the data and tested the predictive model with the remaining tenth of data. Next, we repeated the previous step nine more times by leaving out distinct nine-tenths of the data as training data and a distinct tenth of data as testing data. Finally, we reported the average estimation over all runs by processing the aforementioned regular 10-fold cross-validation for 10 times with distinct batches of data. We estimated the performance of all predictive models using repeated 10-fold cross-validation testing.

## Results

A total of 421 MDD patients (mean age of 43.7 years and 28.7% of males) were retained for the following analyses after we removed the subjects with missing or incomplete data. Table 1 describes the demographic and clinical characteristics of the study population, including 257 antidepressant treatment responders and 164 treatment non-responders. The treatment-response rate in our cohort was 61.0%. Additionally, the remission rate was 33.0%. Six clinical biomarkers were used in the subsequent deep learning analyses, including age at time of consent, sex, marital status (or in a long-term relationship), the number of depressive episodes until time of study enrollment, 21-item HRSD at baseline, and the status of whether the patients had previously attempted suicide. There was a significant difference in the number of depressive episodes (*P* = 0.039) and 21-item HRSD at baseline (*P* = 0.013) between the treatment response and non-response subjects (Table 1). Furthermore, there was a significant difference in 21-item HRSD at baseline (*P* = 0.022) between the remitted and non-remitted subjects (Table 1).

**Table 1:** Demographic and clinical characteristics of study subjects.

**Characteristic**	**All**	**Treatment responder**	**Treatment non-responder**	***P*-value**	**Remission**	**Non-remission**	***P*-value**
No. of subjects (n)	421	257	164		139	282	
Age at time of consent (years)	43.7 ± 14.6	44.7 ± 14.4	41.9 ± 14.9	0.057	43.8 ± 13.6	43.5 ± 15.2	0.849
Sex (male/n; %)	121; 28.7%	79; 30.7%	42; 25.6%	0.257	39; 28.1%	82; 29.1%	0.828
Patient married or in a long-term relationship (n; %)	242; 57.5%	154; 59.9%	87; 53.0%	0.165	80; 57.6%	161; 57.1%	0.928
Number of depressive episodes until time of study enrollment	1.4 ± 1.0	1.4 ± 0.9	1.5 ± 1.2	0.039	1.4 ± 0.7	1.5 ± 1.1	0.250
21-item HRSD at baseline	24.4 ± 5.1	24.9 ± 5.0	23.6 ± 5.2	0.013	23.6 ± 5.1	24.8 ± 5.1	0.022
Suicide attempt (n; %)	12; 2.9%	7; 2.7%	5; 3.0%	0.845	4; 2.9%	8; 2.8%	0.981

*HRSD, Hamilton Rating Scale for Depression*.

*Data are presented as mean ± standard deviation*.

First, we investigated the association between antidepressant treatment response and 4,241,701 SNPs assessed in a GWAS study. None of these SNPs reached the genome-wide significance level (*P* < 1.2 × 10^−8^) after Bonferroni correction for the multiple comparisons. For further investigation in the subsequent deep learning analyses, we identified 10 key SNPs showing an evidence of association with antidepressant treatment responders *per se* with the criterion of a significant level of *P* < 7.5 × 10^−5^ (Table 2). The top-rated SNPs encompass rs4917029 adjacent to the ATP binding cassette subfamily A member 13 (*ABCA13*) gene, rs9419139 adjacent to the BCL2 interacting protein 3 (*BNIP3*) gene, rs704329 in the calcium voltage-gated channel subunit alpha1 E (*CACNA1E*) gene, rs6978272 in the exocyst complex component 4 (*EXOC4*) gene, rs7954376 adjacent to the glutamate ionotropic receptor NMDA type subunit 2B (*GRIN2B*) gene, rs4352778 in the LHFPL tetraspan subfamily member 3 (*LHFPL3*) gene, rs2139423 in the neural EGFL like 1 (*NELL1*) gene, rs2956406 in the NUAK family kinase 1 (*NUAK1*) gene, rs4810894 adjacent to the phosphatidylinositol-3,4,5-trisphosphate dependent Rac exchange factor 1 (*PREX1*) gene, and rs139863958 adjacent to the slit guidance ligand 3 (*SLIT3*) gene. For example, as demonstrated in Table 2 for the rs4810894 SNP of the *PREX1* gene, there was an indication of an association with antidepressant treatment response after adjustment of covariates such as age, sex, and site for the dominant model (OR = 2.70; 95% CI = 1.78–4.10; *P* = 3.20 × 10^−6^). Similarly, there was an indication of an association with antidepressant treatment response among the subjects after adjustment of covariates for the dominant model for the rs7954376 SNP of the *GRIN2B* gene (OR = 0.29; 95% CI = 0.17–0.49; *P* = 3.96 × 10^−6^).

**Table 2:** Odds ratio analysis with odds ratios after adjustment for covariates between treatment response and the top 10 SNPs.

**Gene**	**SNP**	**Chr**	**A1**	**A2**	**Dominant or recessive model**		
					**OR**	**95% CI**	***P***
ABCA13^*^	rs4917029	7	T	C	0.26	0.14–0.50	4.34 × 10^−5^
BNIP3^*^	rs9419139	10	T	C	0.29	0.16–0.51	2.42 × 10^−5^
*CACNA1E*	rs704329	1	A	G	2.27	1.51–3.41	7.46 × 10^−5^
*EXOC4*	rs6978272	7	A	T	0.29	0.17–0.50	6.81 × 10^−6^
GRIN2B^*^	rs7954376	12	T	C	0.29	0.17–0.49	3.96 × 10^−6^
*LHFPL3*	rs4352778	7	T	A	3.22	1.89–5.47	1.63 × 10^−5^
*NELL1*	rs2139423	11	T	G	2.49	1.65–3.78	1.68 × 10^−5^
*NUAK1*	rs2956406	12	C	T	2.39	1.56–3.66	5.89 × 10^−5^
PREX1^*^	rs4810894	20	A	G	2.70	1.78–4.10	3.20 × 10^−6^
SLIT3^*^	rs139863958	5	C	T	0.42	0.27–0.63	4.32 × 10^−5^

*A1, minor allele; A2, major allele; Chr, chromosome; CI, confidence interval; OR, odds ratio*.

*Analysis was obtained after adjustment for covariates including age, sex, and site*.

* *Adjacent gene*.

In addition, we pinpointed 10 key SNPs showing an evidence of association with remission *per se* with the criterion of a significant level of *P* < 9.9 × 10^−5^ (Table 3) for further investigation in the subsequent deep learning analyses. The top-rated SNPs encompass rs11022778 in the aryl hydrocarbon receptor nuclear translocator like (*ARNTL*) gene, rs2724812 in the calcium/calmodulin dependent protein kinase ID (*CAMK1D*) gene, rs12904459 adjacent to the gamma-aminobutyric acid type A receptor beta3 subunit (*GABRB3*) gene, rs35864549 adjacent to the glutamate metabotropic receptor 8 (*GRM8*) gene, rs9878985 in the N-acetylated alpha-linked acidic dipeptidase like 2 (*NAALADL2*) gene, rs483986 in the neurocalcin delta (*NCALD*) gene, rs12046378 adjacent to the phospholipase A2 group IVA (*PLA2G4A*) gene, rs73103153 adjacent to the prokineticin 2 (*PROK2*) gene, rs17134927 in the RNA binding fox-1 homolog 1 (*RBFOX1*) gene, and rs77554113 adjacent to the zinc finger protein 536 (*ZNF536*) gene. For instance, as shown in Table 3 for the rs77554113 SNP of the *ZNF536* gene, there was an indication of an association with remission after adjustment of covariates such as age, sex, and site for the dominant model (OR = 4.81; 95% CI = 2.54–9.13; *P* = 1.49 × 10^−6^). Similarly, there was an indication of an association with remission among the subjects after adjustment of covariates for the dominant model for the rs12904459 SNP of the *GABRB3* gene (OR = 2.53; 95% CI = 1.64–3.90; *P* = 2.58 × 10^−5^).

**Table 3:** Odds ratio analysis with odds ratios after adjustment for covariates between remission and the top 10 SNPs.

**Gene**	**SNP**	**Chr**	**A1**	**A2**	**Dominant or recessive model**		
					**OR**	**95% CI**	***P***
*ARNTL*	rs11022778	11	G	T	2.52	1.59–4.00	9.09 × 10^−5^
*CAMK1D*	rs2724812	10	C	T	3.50	1.88–6.52	7.88 × 10^−5^
GABRB3^*^	rs12904459	15	C	T	2.53	1.64–3.90	2.58 × 10^−5^
GRM8^*^	rs35864549	7	C	A	3.28	1.81–5.94	8.68 × 10^−5^
*NAALADL2*	rs9878985	3	T	C	0.42	0.28–0.65	8.23 × 10^−5^
*NCALD*	rs483986	8	T	A	3.92	2.01–7.62	5.99 × 10^−5^
PLA2G4A^*^	rs12046378	1	A	T	2.36	1.53–3.63	9.56 × 10^−5^
PROK2^*^	rs73103153	3	T	C	2.87	1.70–4.85	8.58 × 10^−5^
*RBFOX1*	rs17134927	16	C	T	2.65	1.66–4.22	4.14 × 10^−5^
ZNF536^*^	rs77554113	19	A	G	4.81	2.54–9.13	1.49 × 10^−6^

*A1, minor allele; A2, major allele; Chr, chromosome; CI, confidence interval; OR, odds ratio*.

*Analysis was obtained after adjustment for covariates including age, sex, and site*.

* *Adjacent gene*.

**Figure 1:**
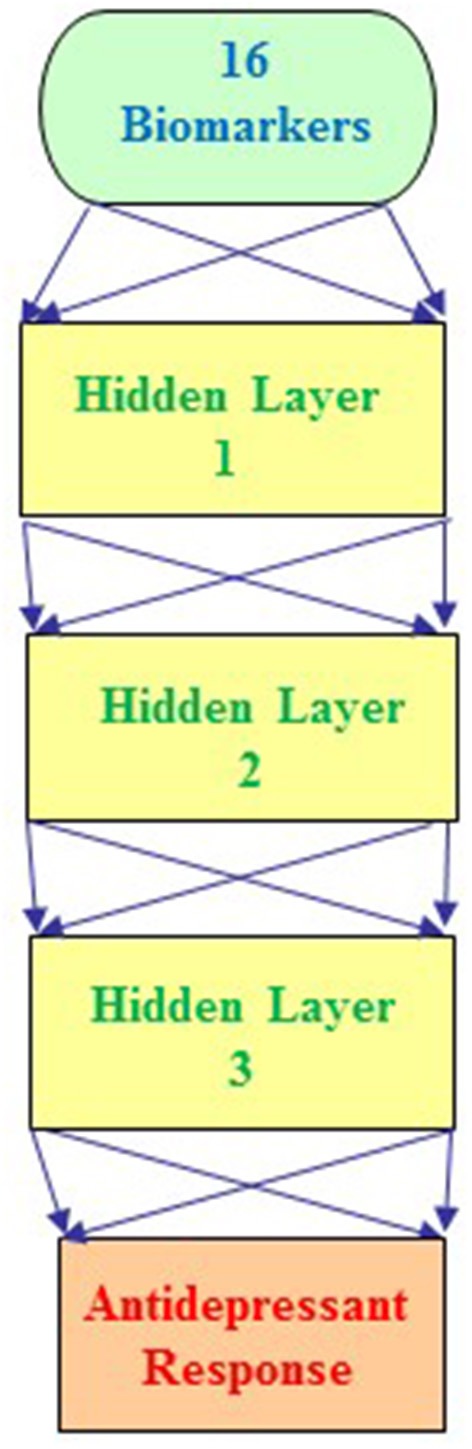
An example architecture of a multilayer feedforward neural network (MFNN) model with 3 hidden layers. The MFNN model contains 16 units in the input layer corresponding to 16 biomarkers (including 10 SNPs and 6 clinical predictors). The MFNN model is configured with 2 units in the output layer corresponding to antidepressant treatment outcome (that is, antidepressant treatment responders and non-responders).

Furthermore, we integrated the aforementioned 6 clinical biomarkers with the top 10 SNPs of treatment response to build the predictive models for antidepressant treatment response by using the deep MFNN framework. Table 4 summarizes the results of repeated 10-fold cross-validation experiments by deep MFNN models and logistic regression using 16 biomarkers including the aforementioned 10 SNPs of treatment response and 6 clinical biomarkers. For deep MFNN models, we performed a series of different architectures containing 1, 2, and 3 hidden layers. Figure 1 shows an example of architecture of the MFNN model with 3 hidden layers. To measure the performance of prediction models, we used the ROC methodology and calculated the AUC, sensitivity, and specificity for these four predictive models using 16 biomarkers. As indicated in Table 4, the average values of AUC for the deep MFNN prediction models of 1, 2, and 3 hidden layers were 0.8211 ± 0.0571, 0.8228 ± 0.0571, and 0.8220 ± 0.0570, respectively. Of all the MFNN prediction models, the deep MFNN model with 2 hidden layers gave better performance than the other two models in terms of AUC. Among all four predictive models, the deep MFNN model with 2 hidden layers performed best, outperforming the logistic regression model (AUC = 0.8168 ± 0.0553) in terms of AUC. For comparison, we also built the predictive models for antidepressant treatment response using only 6 clinical biomarkers. The models with 16 biomarkers performed better than the ones with only 6 clinical biomarkers (Table 4).

**Table 4:** The results of repeated 10-fold cross-validation experiments for predicting treatment response using multilayer feedforward neural networks (MFNNs) and logistic regression with 16 biomarkers and 6 clinical biomarkers only.

**Algorithm**	**AUC**	**Sensitivity**	**Specificity**	**Number of biomarkers**
MFNN with 1 hidden layer	0.8211 ± 0.0571	0.7496 ± 0.0579	0.6775 ± 0.0731	16
MFNN with 2 hidden layers	0.8228 ± 0.0571	0.7546 ± 0.0619	0.6922 ± 0.0765	16
MFNN with 3 hidden layers	0.8220 ± 0.0570	0.7535 ± 0.0611	0.6951 ± 0.0731	16
Logistic Regression	0.8168 ± 0.0553	0.7493 ± 0.0626	0.7066 ± 0.0785	16
MFNN with 1 hidden layer	0.5597 ± 0.0808	0.6081 ± 0.0113	0.3919 ± 0.0113	6
MFNN with 2 hidden layers	0.5606 ± 0.0836	0.6081 ± 0.0113	0.3919 ± 0.0113	6
MFNN with 3 hidden layers	0.5571 ± 0.0788	0.6081 ± 0.0113	0.3919 ± 0.0113	6
Logistic Regression	0.5374 ± 0.0762	0.5881 ± 0.0432	0.4112 ± 0.0418	6

*AUC, the area under the receiver operating characteristic curve*.

*Data are presented as mean ± standard deviation*.

Moreover, we combined the aforementioned 6 clinical biomarkers with the top 10 SNPs of remission to construct the predictive models for remission by using the deep MFNN framework. Table 5 summarizes the results of repeated 10-fold cross-validation experiments by deep MFNN models and logistic regression using 16 biomarkers including the aforementioned 10 SNPs of remission and 6 clinical biomarkers. For deep MFNN models, we achieved a series of different architectures containing 1, 2, and 3 hidden layers. As indicated in Table 5, the average values of AUC for the deep MFNN prediction models of 1, 2, and 3 hidden layers were 0.8042 ± 0.0729, 0.8047 ± 0.0727, and 0.8060 ± 0.0722, respectively. Of all the MFNN prediction models, the deep MFNN model with 3 hidden layers gave better performance than the other two models in terms of AUC. Among all four predictive models, the deep MFNN model with 3 hidden layers performed best, outperforming the logistic regression model (AUC = 0.7985 ± 0.0772) in terms of AUC. For comparison, we also built the predictive models for remission using only 6 clinical biomarkers. The models with 16 biomarkers performed better than the ones with only 6 clinical biomarkers (Table 5).

**Table 5:** The results of repeated 10-fold cross-validation experiments for predicting remission using multilayer feedforward neural networks (MFNNs) and logistic regression with 16 biomarkers and 6 clinical biomarkers only.

**Algorithm**	**AUC**	**Sensitivity**	**Specificity**	**Number of biomarkers**
MFNN with 1 hidden layer	0.8042 ± 0.0729	0.7689 ± 0.0579	0.6580 ± 0.0839	16
MFNN with 2 hidden layers	0.8047 ± 0.0727	0.7734 ± 0.0593	0.6643 ± 0.0832	16
MFNN with 3 hidden layers	0.8060 ± 0.0722	0.7732 ± 0.0583	0.6623 ± 0.0853	16
Logistic Regression	0.7985 ± 0.0772	0.7722 ± 0.0645	0.6753 ± 0.0932	16
MFNN with 1 hidden layer	0.6089 ± 0.0848	0.6698 ± 0.0073	0.3302 ± 0.0073	6
MFNN with 2 hidden layers	0.6135 ± 0.0871	0.6698 ± 0.0073	0.3302 ± 0.0073	6
MFNN with 3 hidden layers	0.6116 ± 0.0872	0.6698 ± 0.0073	0.3302 ± 0.0073	6
Logistic Regression	0.5922 ± 0.0878	0.6501 ± 0.0292	0.3330 ± 0.0290	6

*AUC, the area under the receiver operating characteristic curve*.

*Data are presented as mean ± standard deviation*.

Finally, the GMDR analysis was used to assess SNP-SNP interactions between the top 10 SNPs in antidepressant treatment response including age, sex, and site as covariates. Table 6 summarizes the results obtained from GMDR analysis for SNP-SNP interaction models with covariate adjustment. As shown in Table 6, there was a significant SNP-SNP interaction involving *BNIP3* rs9419139 and *PREX1* rs4810894 (*P* < 0.001) in influencing antidepressant treatment response. We also assessed SNP-SNP interactions between the top 10 SNPs in remission including age, sex, and site as covariates. As shown in Table 6, there was a significant SNP-SNP interaction involving *ARNTL* rs11022778 and *ZNF536* rs77554113 (P < 0.001) in influencing remission.

**Table 6:** SNP-SNP interaction models identified by the GMDR method with adjustment for age, sex, and site.

**Phenotype**	**SNP-SNP interaction model**	**Testing accuracy (%)**	***P*-value**
Antidepressant treatment response	*BNIP3* rs9419139, *PREX1* rs4810894	66.92	*P* < 0.001
Remission	*ARNTL* rs11022778, *ZNF536* rs77554113	59.69	*P* < 0.001

*GMDR, generalized multifactor dimensionality reduction*.

*P-value was based on 1,000 permutations. Analysis was obtained after adjustment for covariates including age, sex, and site*.

## Discussion

Our analysis is the first study to date to leverage deep learning for building predictive models of antidepressant treatment outcome among Taiwanese MDD individuals with the GWAS data. In this study, we pinpointed that the deep MFNN model with 2 hidden layers outperformed the logistic regression model as well as other predictive models in terms of AUC for distinguishing antidepressant treatment responders and non-responders in MDD. We also found that the deep MFNN model with three hidden layers exceeded the logistic regression model as well as other predictive models in terms of AUC for predicting remission in MDD. Additionally, we identified 26 predictive variables of antidepressants, including 6 clinical biomarkers, 10 SNPs for treatment response, and 10 SNPs for remission. The 6 clinical biomarkers encompass the patient's age at time of consent, sex, marital status (or in a long-term relationship), the number of depressive episodes until time of study enrollment, 21-item HRSD at baseline, and the status of suicide attempts. By using the GWAS data, we further tracked down the top 10 SNPs showing an evidence of association with antidepressant treatment response as well as remission, respectively. Moreover, we combined the 6 clinical predictors with the 10 genetic variants to establish the predictive models of antidepressant treatment outcome as well as remission by using the deep MFNN framework. Our data also indicated that our deep MFNN models may provide a suitable approach to create predictive models for forecasting antidepressant treatment response as well as remission with clinically meaningful accuracy. Therefore, our deep MFNN approach is a proof of concept of a deep learning predictive tool for antidepressant efficacy prior to antidepressant treatment.

In the present study, we found that 10 potential SNPs (including *ABCA13* rs4917029, *BNIP3* rs9419139, *CACNA1E* rs704329, *EXOC4* rs6978272, *GRIN2B* rs7954376, *LHFPL3* rs4352778, *NELL1* rs2139423, *NUAK1* rs2956406, *PREX1* rs4810894, and *SLIT3* rs139863958) may play an important role in the modulation of antidepressant treatment response in a Taiwanese population by using a GWAS study. However, to our knowledge, no studies have been implicated these SNPs for drug efficacy of antidepressants. The functional relevance of the effects of these SNPs on antidepressant treatment response remains to be elucidated. The *BNIP3* gene is located on chromosome 10q26.3 and encodes a mitochondrial protein which is implicated in several functions linked to antidepressive effects as well as the action of antidepressants (34). The *ABCA13, PREX1*, and *SLIT3* genes have been suggested to link with MDD (35–37). The *CACNA1E* gene has been shown to be associated with bipolar disorder (38). The *GRIN2B* and *EXOC4* genes have been found to be in relation to schizophrenia (39, 40). The *LHFPL3, NELL1*, and *NUAK1* gene might be connected to the synapse function, mental development delay, and neuropsychiatric disorders, respectively (41–43).

Moreover, we identified 10 potential SNPs (including *ARNTL* rs11022778, *CAMK1D* rs2724812, *GABRB3* rs12904459, *GRM8* rs35864549, *NAALADL2* rs9878985, *NCALD* rs483986, *PLA2G4A* rs12046378, *PROK2* rs73103153, *RBFOX1* rs17134927, and *ZNF536* rs77554113) to be associated with remission in a Taiwanese population by using a GWAS study. The *ARNTL* gene, located on chromosome 11p15.3, is one of the clock genes, representing the hallmark of circadian rhythms that have been shown to influence the behavioral effects of psychoactive drugs (44). *ARNTL* rs11022778 SNP has also been suggested to be involved to in the susceptibility of mood disorders (both MDD and bipolar disorders) and suicide attempts (45, 46). The *GRM8, PLA2G4A*, and *PROK2* genes were found to be associated with MDD (47–49). The *NCALD* and *ZNF536* genes have been shown to be linked with bipolar disorder (50, 51). The *CAMK1D, GABRB3*, and *RBFOX1* genes have been suggested to contribute to schizophrenia physiopathology (52–54). The *NAALADL2* gene has been implicated to cause autism spectrum disorder, a neurodevelopmental disorder (55).

On another note, our deep MFNN framework utilized 6 clinical biomarkers encompassing demographics data (such as age, sex, marital status), total scores on baseline severity measures (such as 21-item HRSD at baseline), recurrent episodes (that is, the number of depressive episodes until time of study enrollment), and the status of suicide attempts. These 6 clinical biomarkers have been previously tested as predictors for predicting possible antidepressant treatment outcome in MDD in other studies (17, 56). However, to the best of our knowledge, no prior studies have been evaluated for drug efficacy of antidepressants by combining these clinical predictors with the SNP information. There are more potential clinical biomarkers such as body mass index, MDD subtypes, the age of onset of MDD, social support, previous antidepressant treatments, and early life stressful events that could possibly influence antidepressants treatment response (17, 56). However, our study did not comprise these clinical biomarkers due to lack of data.

Remarkably, another intriguing finding was that we further inferred the epistatic effects between *BNIP3* rs9419139 and *PREX1* rs4810894 in influencing antidepressant treatment response as well as the epistatic effects between *ARNTL* rs11022778 and *ZNF536* rs77554113 in influencing remission by using the GMDR approach. To our knowledge, no other study has been conducted to weigh SNP-SNP interactions between these SNPs. The *ARNTL* and *BNIP3* gene have been implicated to influence psychoactive drugs and antidepressants (34, 44). The *PREX1* and *ZNF536* genes have been shown to be associated with MDD and bipolar disorder, respectively (37, 50). The biological mechanisms of these SNP-SNP interactions on antidepressant treatment outcome remain to be elucidated.

This study has both limitations and strengths. The major weakness is that the novel findings related to the selected SNPs were not validated by other independent cohorts and the MFNN models were not verified by an independent cohort or dataset. Both the sensitivity and specificity of the model were also not adequate enough to predict treatment response in clinical practice. In addition, our study was carried out using four different SSRI antidepressants and some patients had concomitant use of alprazolam, lorazepam or clonazepam for insomnia. Moreover, the present study was underpowered because of limited sample size, and few replications of the effects and uncertain biological mechanisms were found for some selected SNPs on antidepressant treatment outcome. Therefore, our findings warrant much more research to evaluate whether the observations are reproducible in various ethnic populations (57). The use of SNPs in this study serves as a strategy for building the MFNN models, and these selected SNPs will need to be replaced with significant SNPs from much larger and more rigorous studies in the future. Future clinical trials are needed to facilitate a comprehensive assessment of the predictive models for antidepressant treatment outcome with different ethnic populations (57). By contrast, the main strength of our work is that we utilized rigorously phenotyped antidepressant response in MDD patients to assess influential SNPs among the investigated genes.

## Conclusions

In conclusion, we carried out deep learning predictive models for assessing antidepressant treatment outcome in Taiwanese subjects. Our findings demonstrate that our deep MFNN framework may provide a plausible way to build predictive models for estimating antidepressant treatment response with clinically meaningful accuracy. Thus, we could anticipate that the results of our studies could be generalized for genomics studies of personalized medicine in predicting treatment response for human disorders and could be employed to establish molecular diagnostic and prognostic tools over the next few years. It is essential to evolve further understandings into the role of these deep learning predictive models investigated in this study by using independent replication studies with larger sample sizes.

## Ethics statement

This study was approved by the Institutional Review Board of Taipei Veterans General Hospital (VGHIRB No.: 2014-06-001B) and complies with the Declaration of Helsinki. Informed written consent was obtained from all participants.

## Author contributions

Study conception and design: EL, P-HK, and S-JT. Acquisition of data: P-HK, Y-LL, YY, and AY. Analysis and interpretation of data: EL and S-JT. Draft manuscript: EL. All authors read and approved the final manuscript.

### Conflict of interest statement

The authors declare that the research was conducted in the absence of any commercial or financial relationships that could be construed as a potential conflict of interest.
